# Supplementary figures and images for: The hidden anatomy of paranasal sinuses reveals biogeographically distinct morphotypes in the nine-banded armadillo (Dasypus novemcinctus)
Source: PeerJ. 2017 Aug 15;5:e3593. doi: 10.7717/peerj.3593 (PMC5562141; doi:10.7717/peerj.3593)

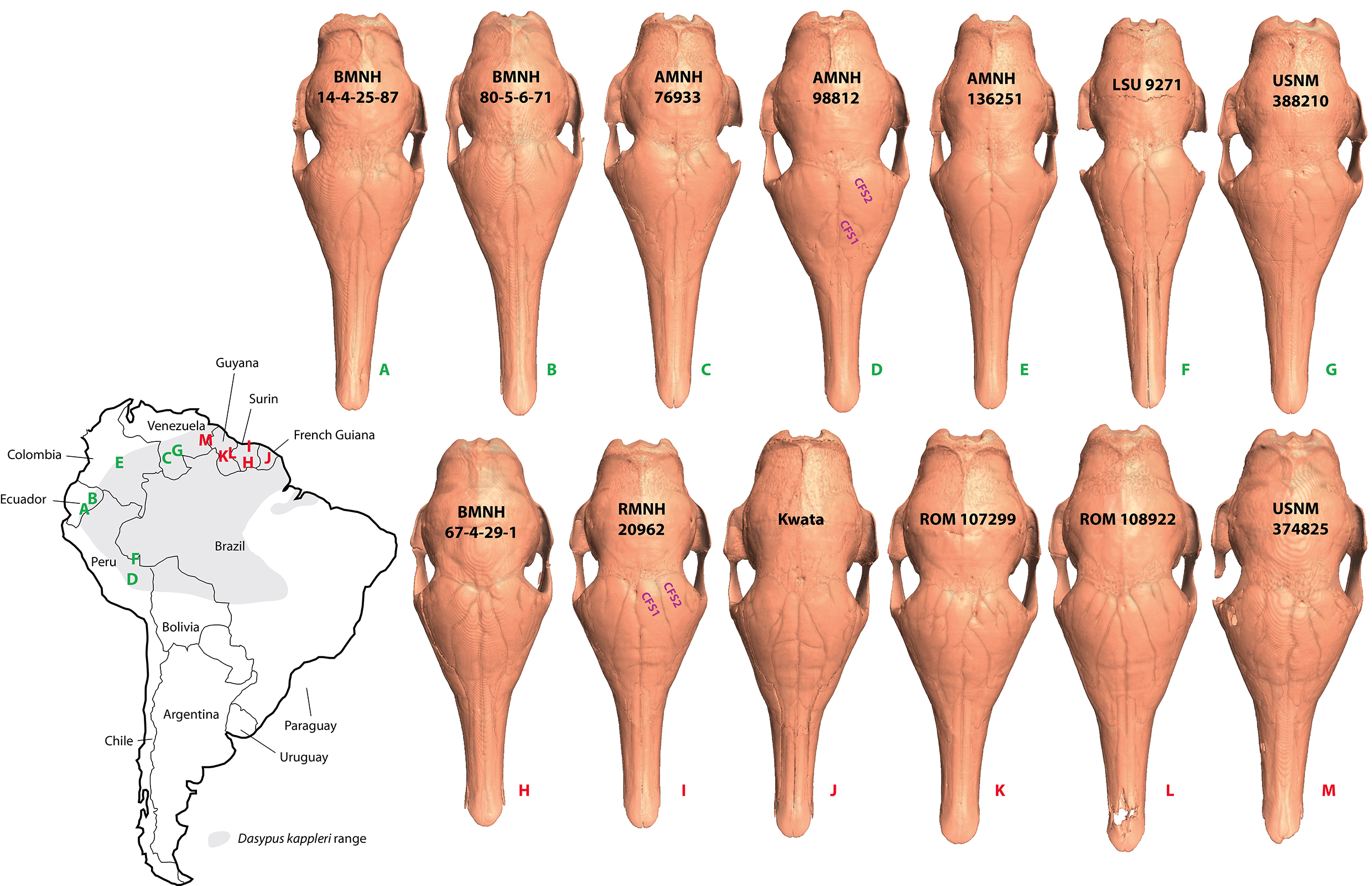

Supplement: Figure S1 — Skulls not to scale. [file peerj-05-3593-s001.jpg]

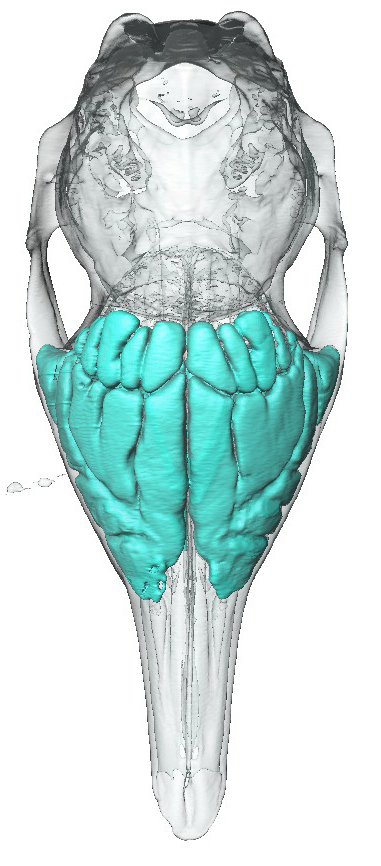

Supplement: Data S1 [file peerj-05-3593-s003.zip › all_sinuses_dorsal/Dasypus_nov_BMNH_26_1_12_22_SnapshotB.jpg]

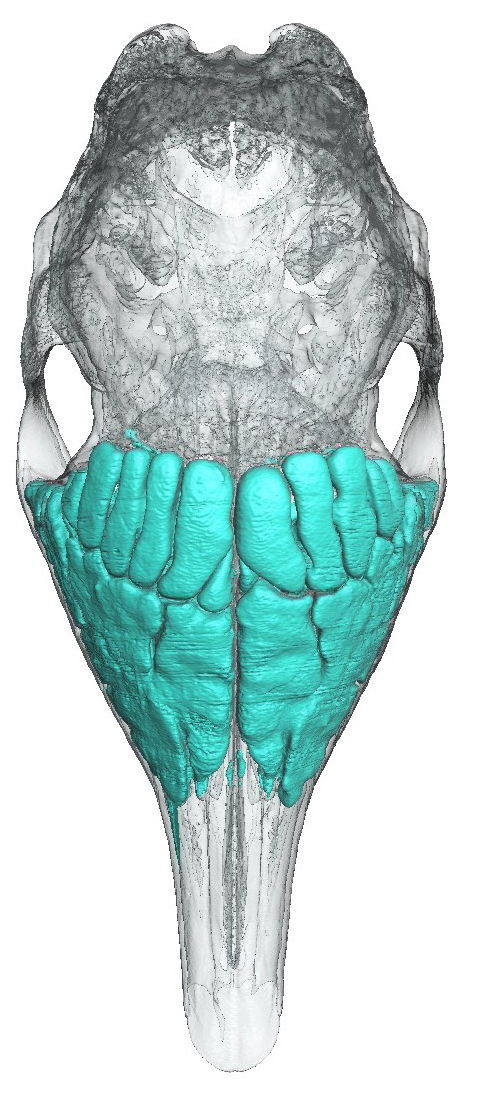

Supplement: Data S1 [file peerj-05-3593-s003.zip › all_sinuses_dorsal/211668_dorsal_trans.jpg]

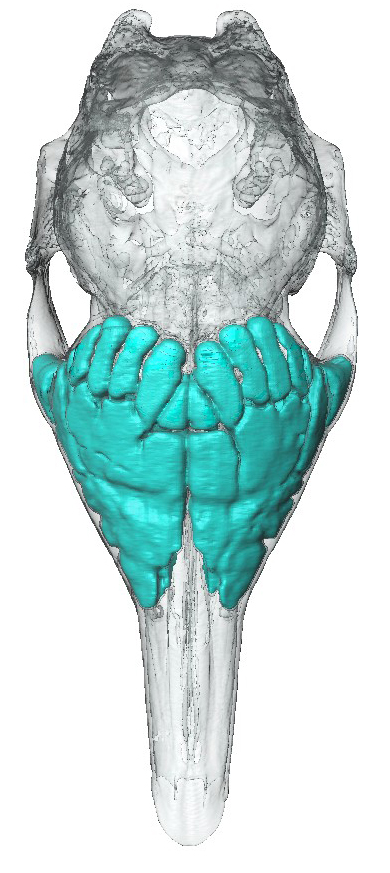

Supplement: Data S1 [file peerj-05-3593-s003.zip › all_sinuses_dorsal/USNM283467_SnapshotB.jpg]

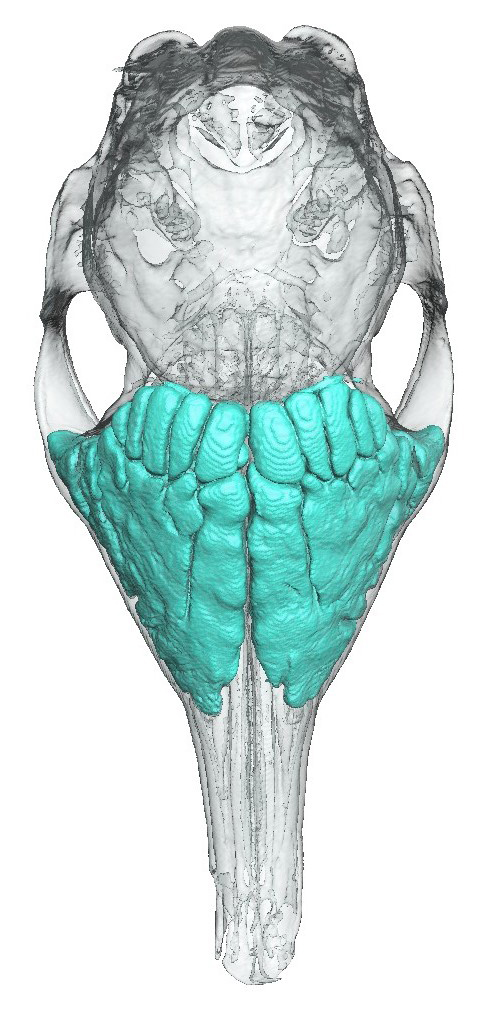

Supplement: Data S1 [file peerj-05-3593-s003.zip › all_sinuses_dorsal/75896_dorsal_trans - Copie.jpg]

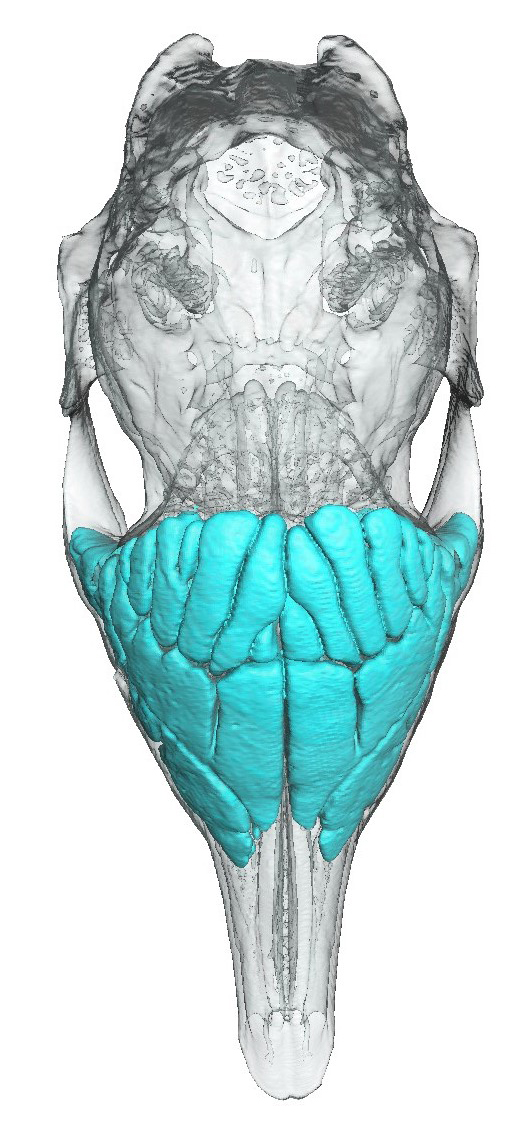

Supplement: Data S1 [file peerj-05-3593-s003.zip › all_sinuses_dorsal/133329_dorsal_trans.jpg]

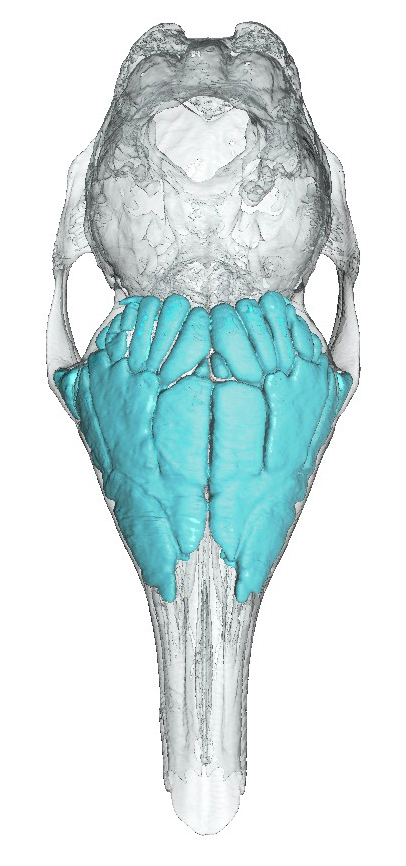

Supplement: Data S1 [file peerj-05-3593-s003.zip › all_sinuses_dorsal/133338_dorsal_trans2.jpg]

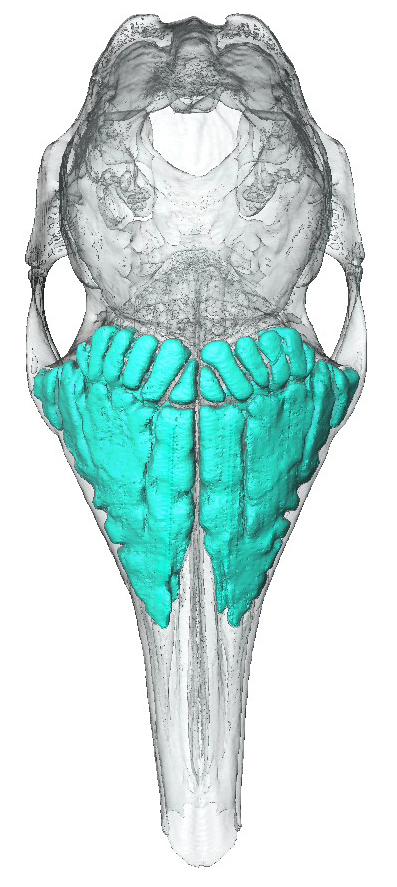

Supplement: Data S1 [file peerj-05-3593-s003.zip › all_sinuses_dorsal/AMNH133265OK_dorsal_transp.jpg]

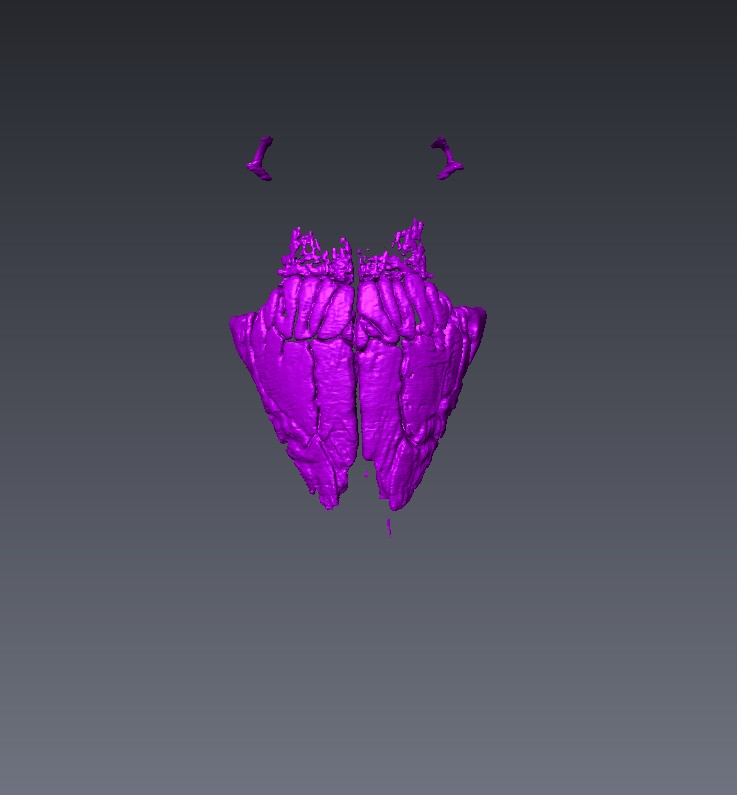

Supplement: Data S1 [file peerj-05-3593-s003.zip › all_sinuses_dorsal/das_nov_mhng_964-67_brazil.jpg]

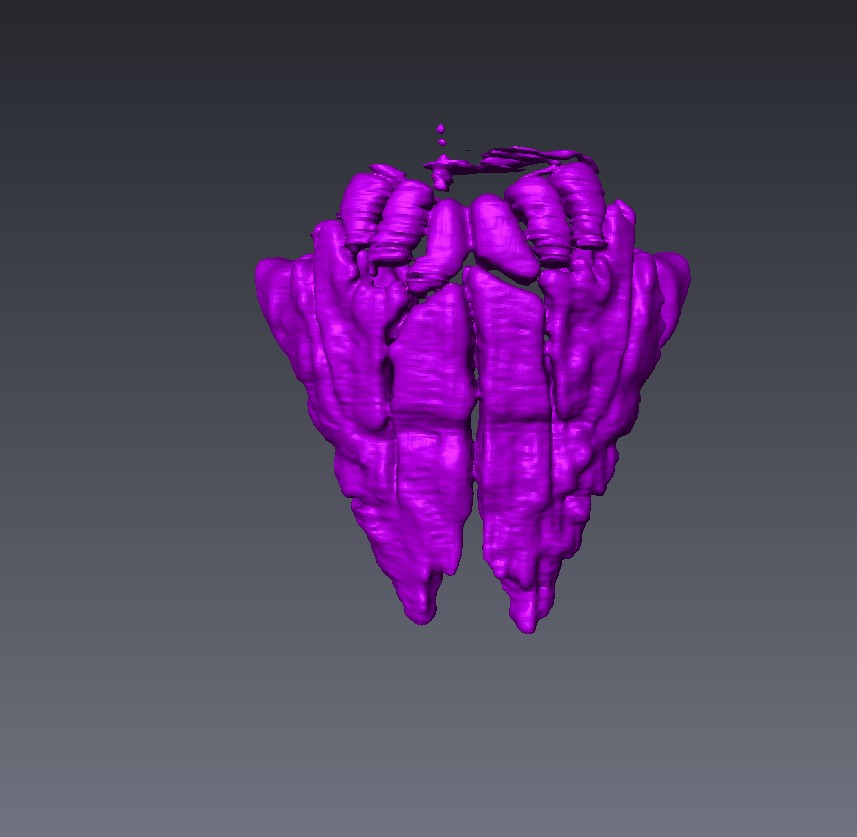

Supplement: Data S1 [file peerj-05-3593-s003.zip › all_sinuses_dorsal/Das_nov_mnhn_2006-565_brazil_para_female.jpg]

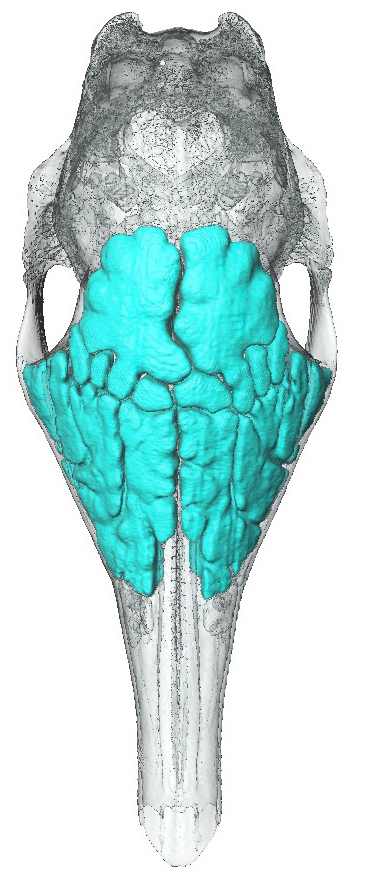

Supplement: Data S1 [file peerj-05-3593-s003.zip › all_sinuses_dorsal/Dasypus_AP207_dorsal_transp.jpg]

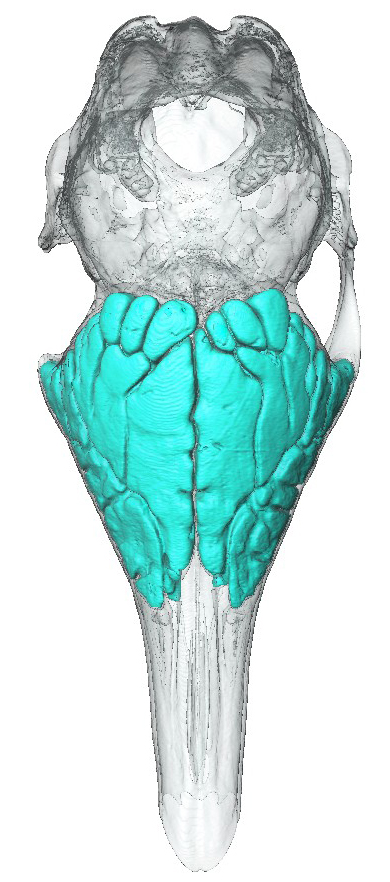

Supplement: Data S1 [file peerj-05-3593-s003.zip › all_sinuses_dorsal/LH_dasypus_nov_3-9-4-102_dorsal_transp.jpg]

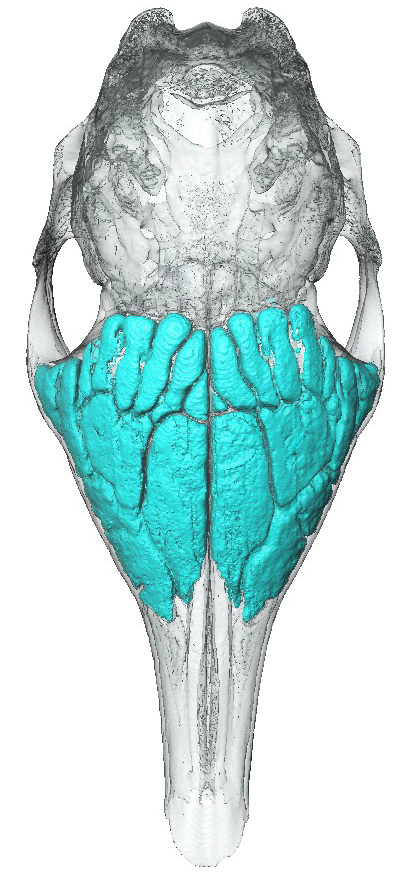

Supplement: Data S1 [file peerj-05-3593-s003.zip › all_sinuses_dorsal/Snapshot_AMNH365_dorsal_transp.jpg]

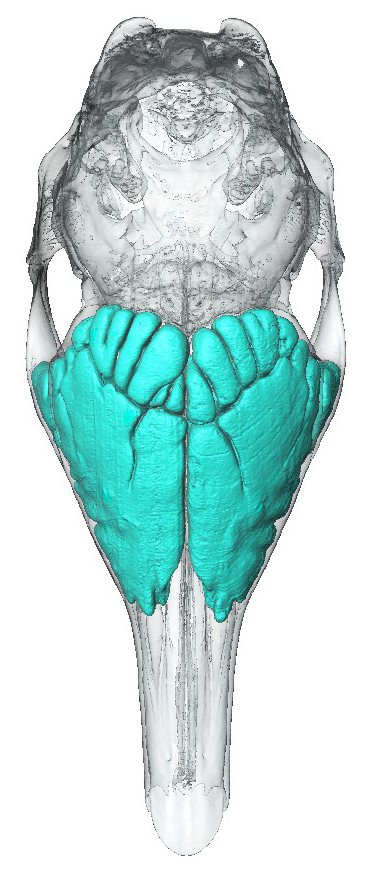

Supplement: Data S1 [file peerj-05-3593-s003.zip › all_sinuses_dorsal/snapshot_Dasypus_nov_AMNH93116_dorsal_transp.jpg]

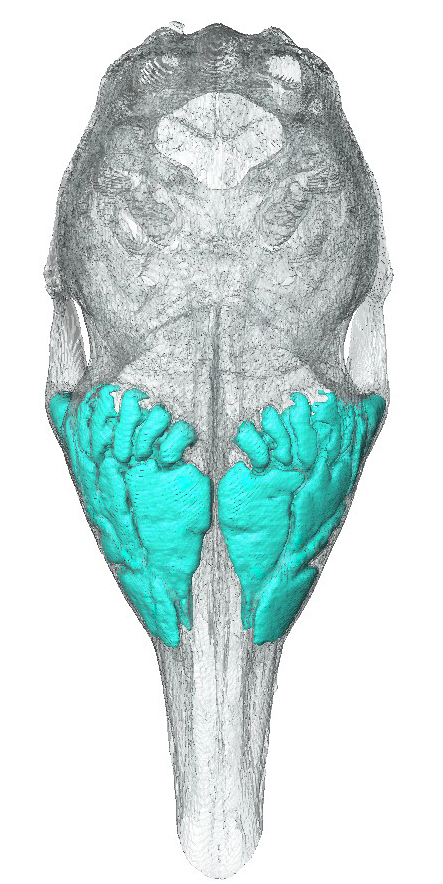

Supplement: Data S1 [file peerj-05-3593-s003.zip › all_sinuses_dorsal/snapshot_Dasypus_nov_AMNH133259_dorsal_transp.jpg]

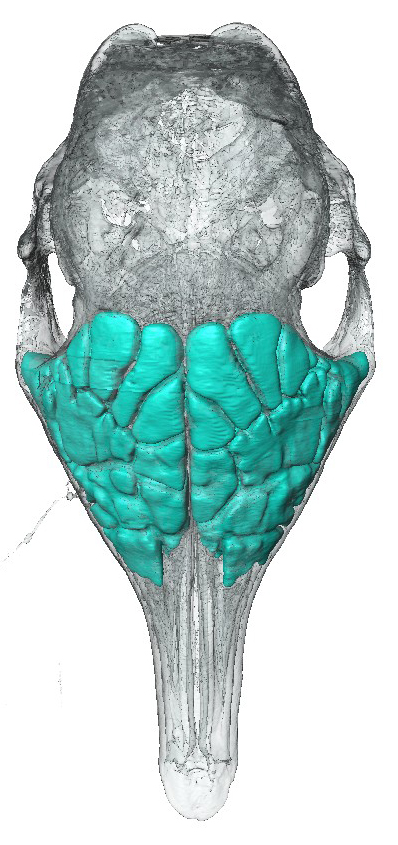

Supplement: Data S1 [file peerj-05-3593-s003.zip › all_sinuses_dorsal/LH_dasypus_nov_98-10-3-23_SnapshotB.jpg]

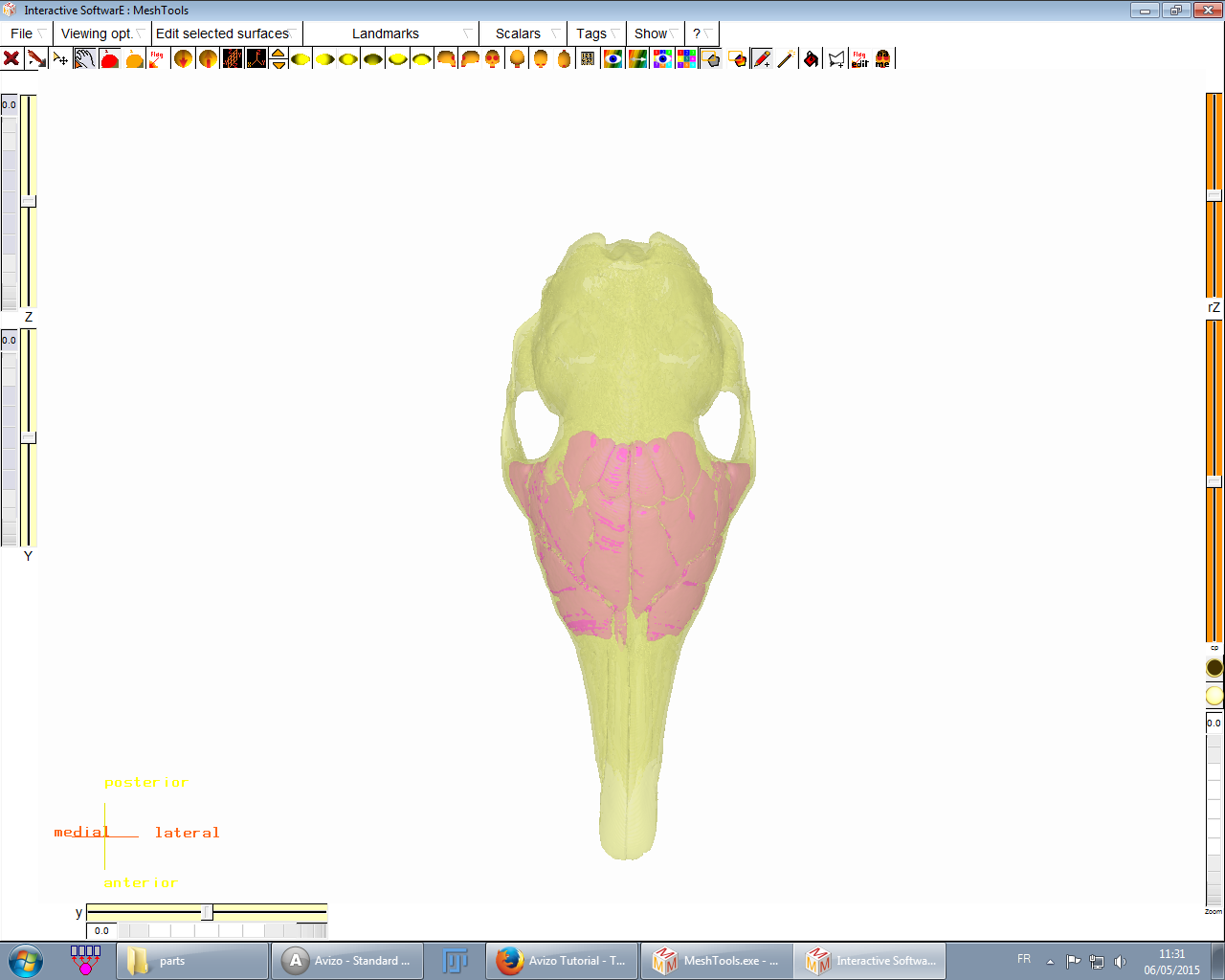

Supplement: Data S1 [file peerj-05-3593-s003.zip › all_sinuses_dorsal/14663_face.png]

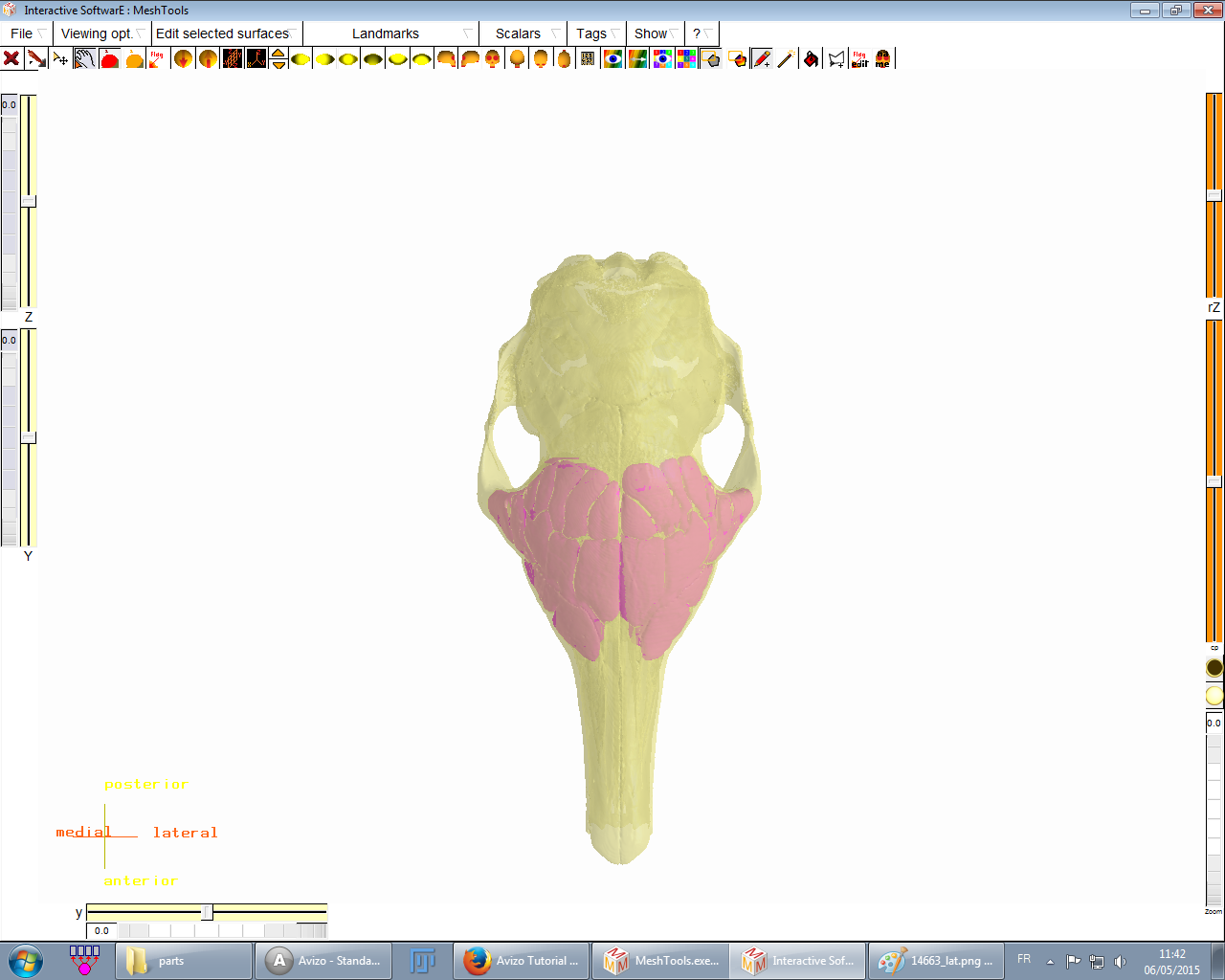

Supplement: Data S1 [file peerj-05-3593-s003.zip › all_sinuses_dorsal/37356_face.png]

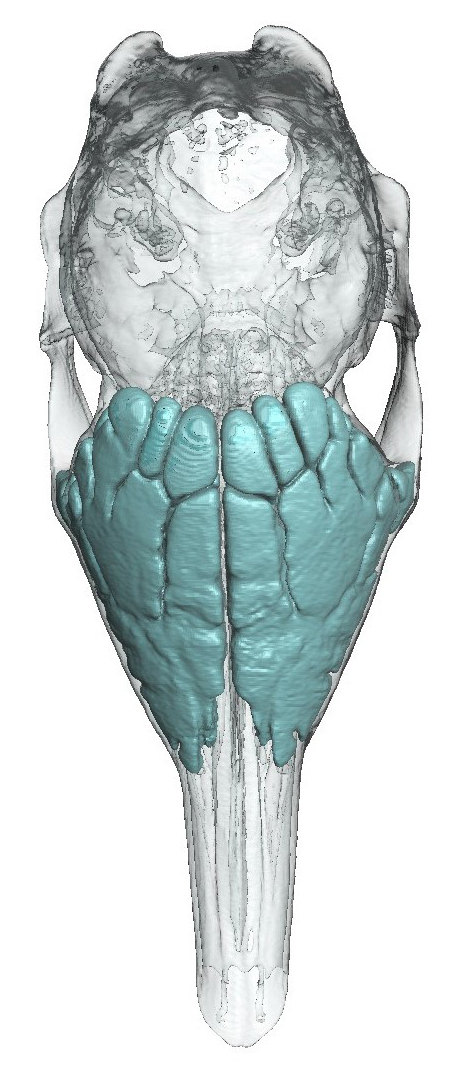

Supplement: Data S1 [file peerj-05-3593-s003.zip › all_sinuses_dorsal/136252_dorsal_trans.jpg]

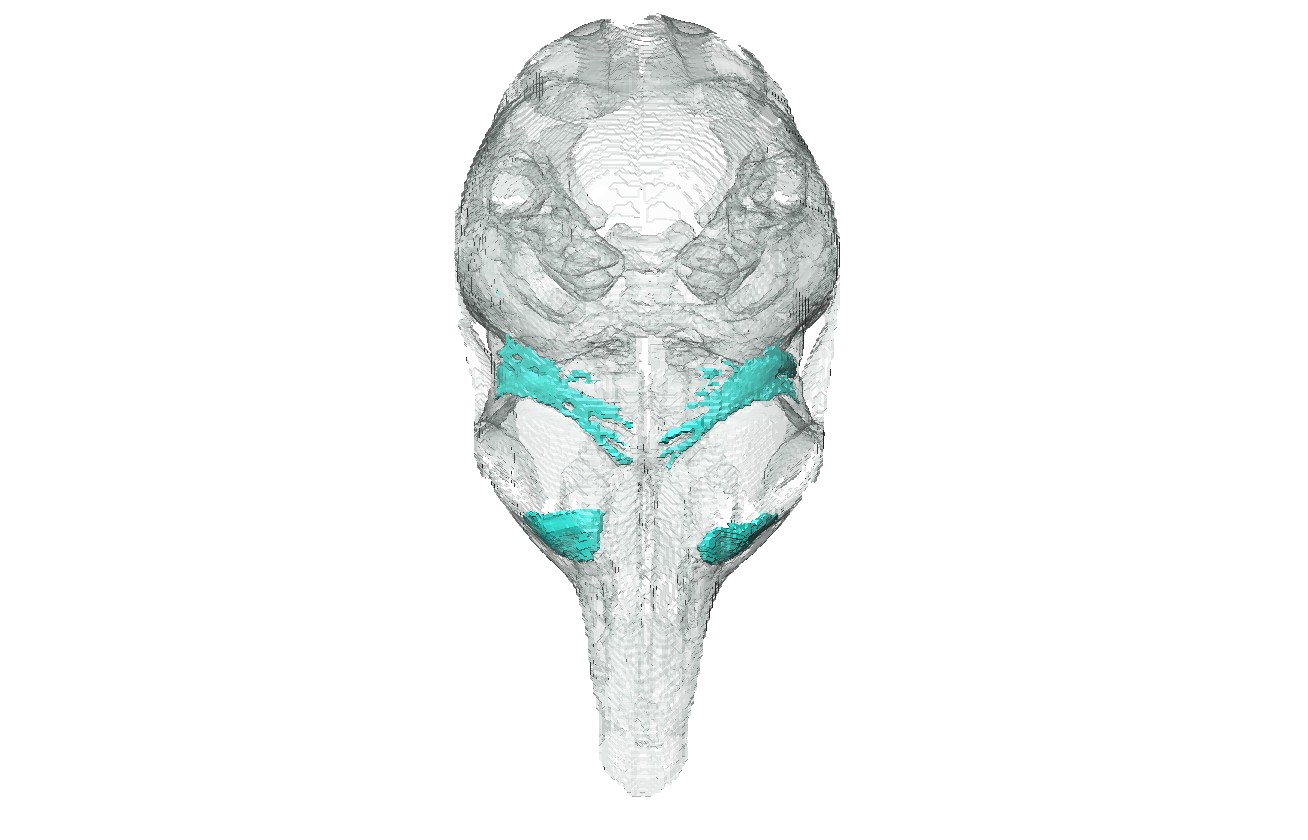

Supplement: Data S1 [file peerj-05-3593-s003.zip › all_sinuses_dorsal/AMNH33150_JUV_dorsal_transp.jpg]

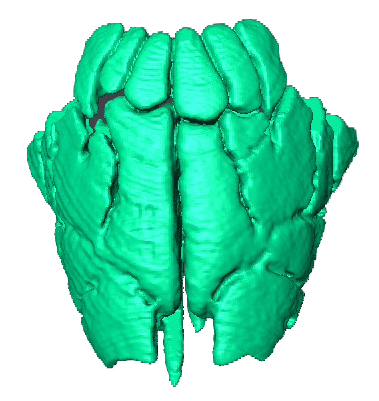

Supplement: Data S1 [file peerj-05-3593-s003.zip › all_sinuses_dorsal/das_nov_amnh_14663_colombie_sinus2.jpg]

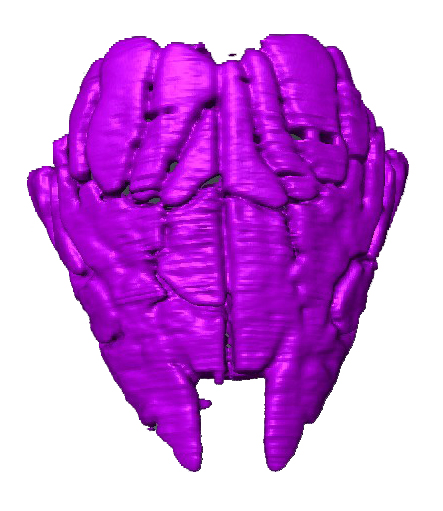

Supplement: Data S1 [file peerj-05-3593-s003.zip › all_sinuses_dorsal/das_nov_amnh_32356_male_colombie_sinus2.jpg]

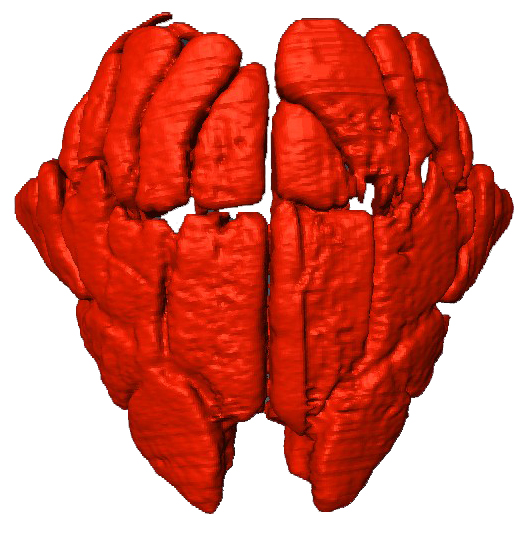

Supplement: Data S1 [file peerj-05-3593-s003.zip › all_sinuses_dorsal/das_nov_amnh_37356_femelle_colombie_sinus2.jpg]

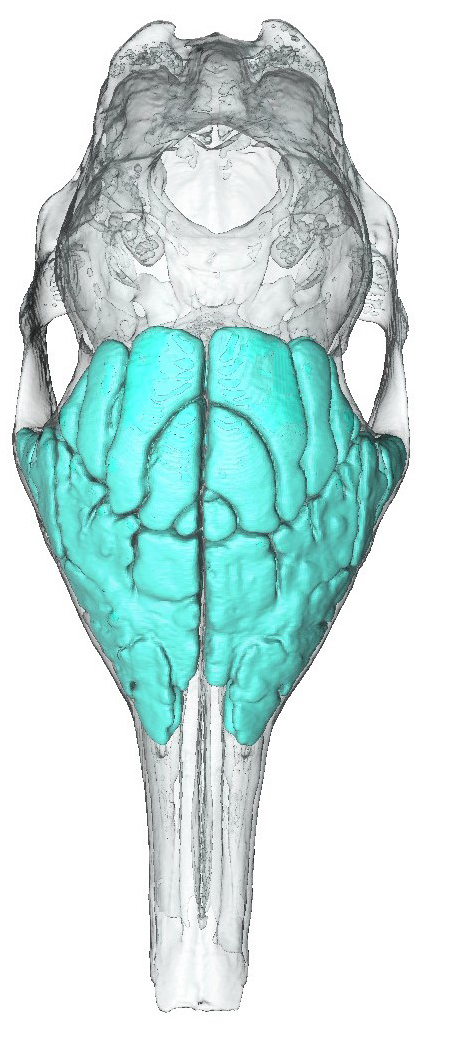

Supplement: Data S1 [file peerj-05-3593-s003.zip › all_sinuses_dorsal/LSU_15762_Costa_Rica_dorsal_trans.jpg]

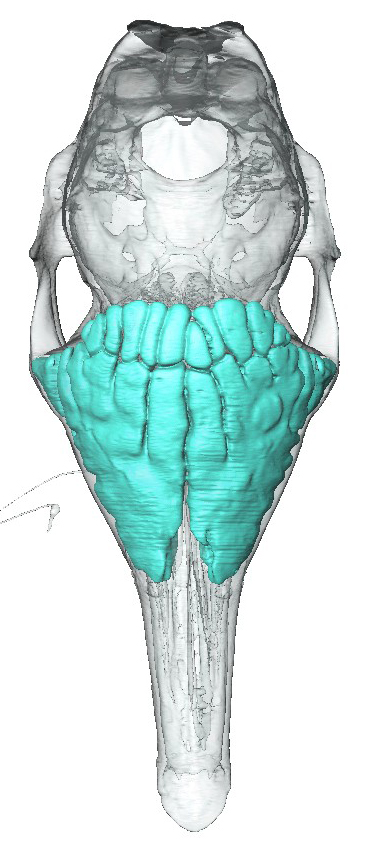

Supplement: Data S1 [file peerj-05-3593-s003.zip › all_sinuses_dorsal/LH_dasypus_nov_14-4-25-86_SnapshotB.jpg]

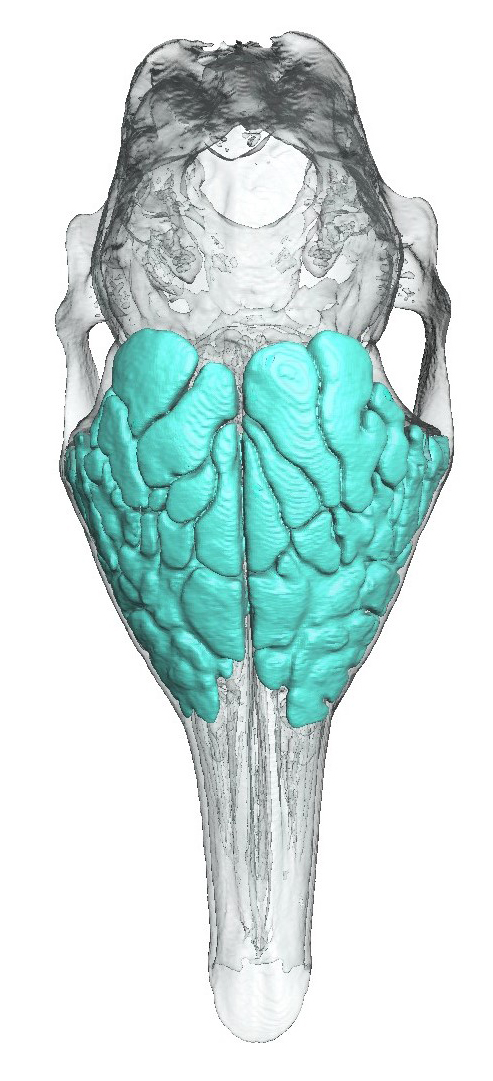

Supplement: Data S1 [file peerj-05-3593-s003.zip › all_sinuses_dorsal/40984_dorsal_trans.jpg]

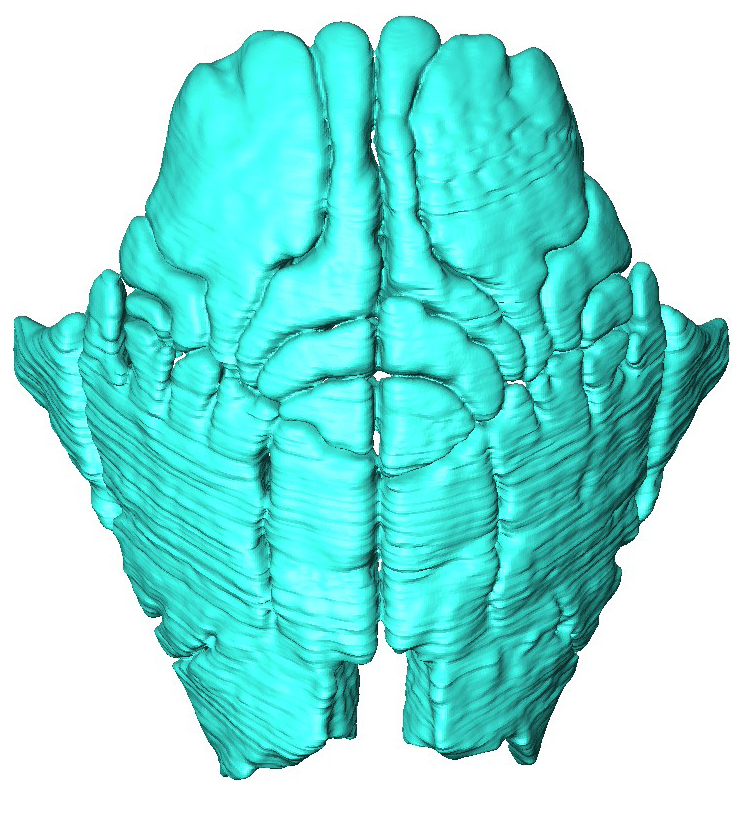

Supplement: Data S1 [file peerj-05-3593-s003.zip › all_sinuses_dorsal/Dasypus_MNHN2001-1317_dorsal_sinuses.jpg]

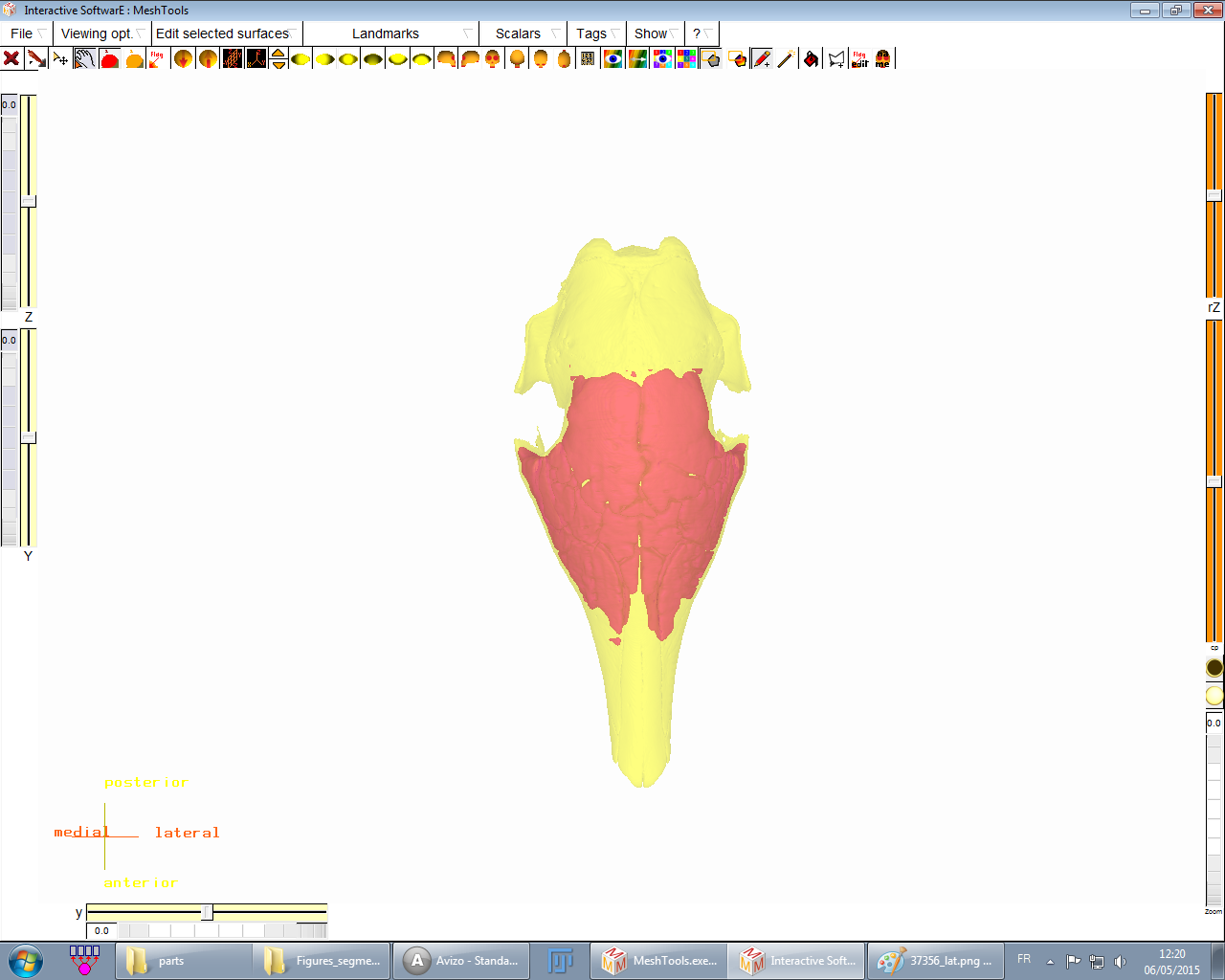

Supplement: Data S1 [file peerj-05-3593-s003.zip › all_sinuses_dorsal/1996-587_face.png]

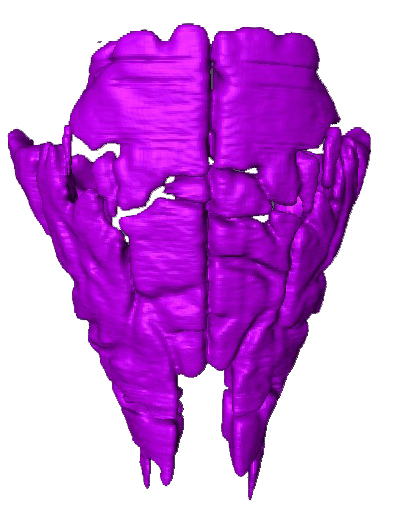

Supplement: Data S1 [file peerj-05-3593-s003.zip › all_sinuses_dorsal/Das_nov_mnhn_1995-953_GF_male_sub2.jpg]

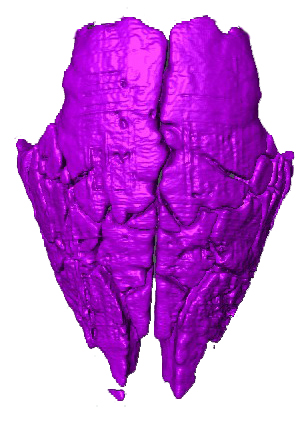

Supplement: Data S1 [file peerj-05-3593-s003.zip › all_sinuses_dorsal/Das_nov_mnhn_1996-587_GF2.jpg]

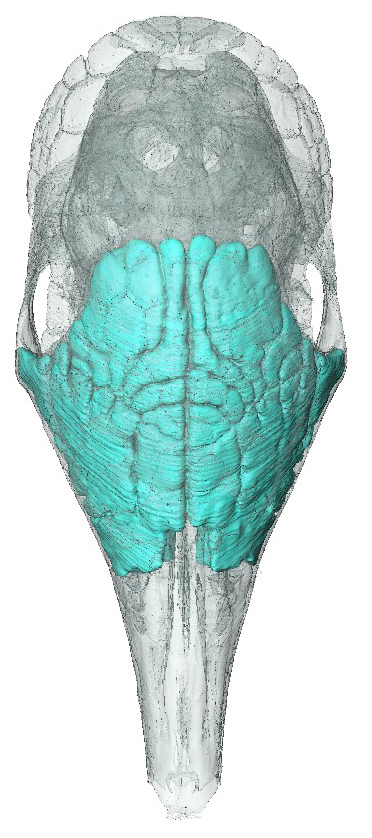

Supplement: Data S1 [file peerj-05-3593-s003.zip › all_sinuses_dorsal/Dasypus_guyan_dorsal_transp3.jpg]

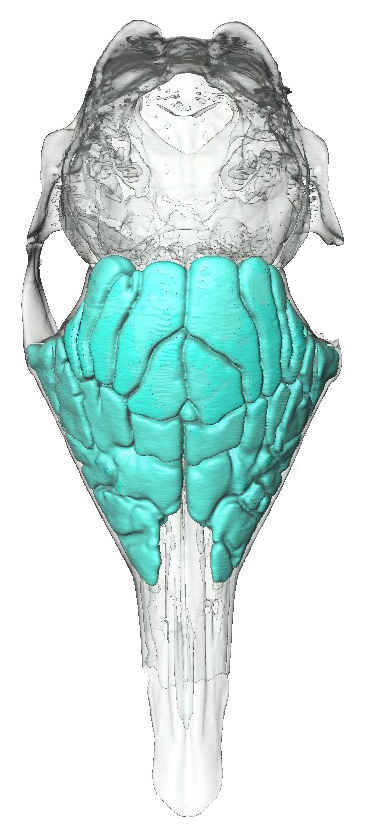

Supplement: Data S1 [file peerj-05-3593-s003.zip › all_sinuses_dorsal/Dasypus_nov_65-5-18-2T_SnapshotB.jpg]

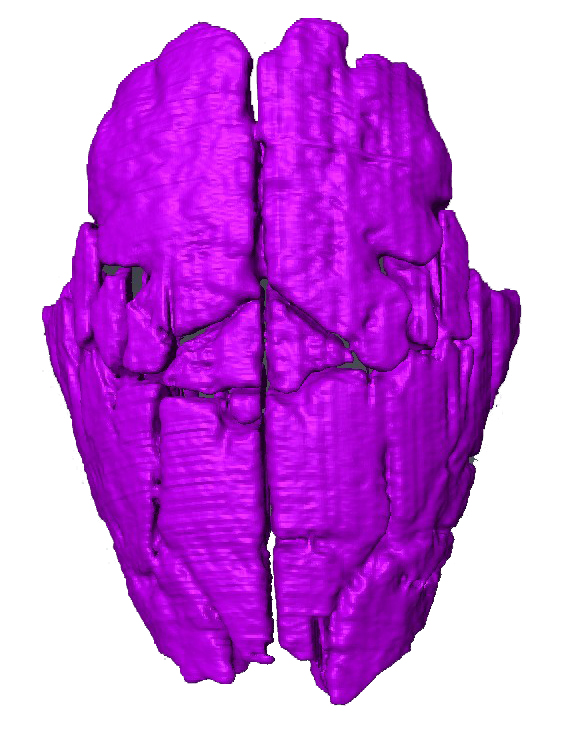

Supplement: Data S1 [file peerj-05-3593-s003.zip › all_sinuses_dorsal/Das_nov_rom_32275_guyana_male2.jpg]

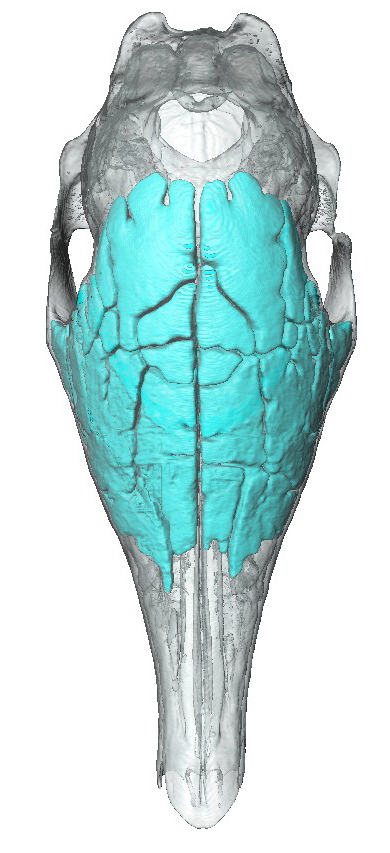

Supplement: Data S1 [file peerj-05-3593-s003.zip › all_sinuses_dorsal/339668_dorsal_trans.jpg]

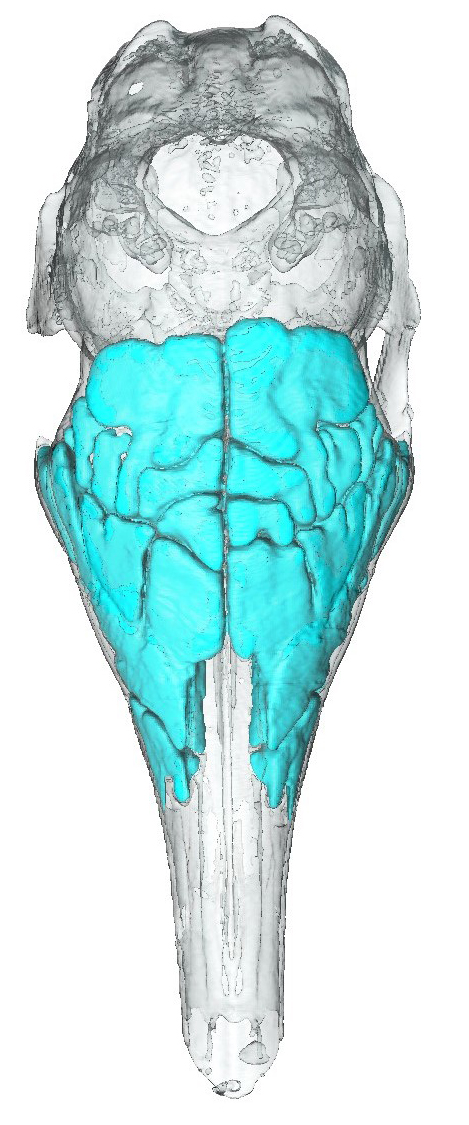

Supplement: Data S1 [file peerj-05-3593-s003.zip › all_sinuses_dorsal/AMNH42883_Guyana_dorsal_trans.jpg]

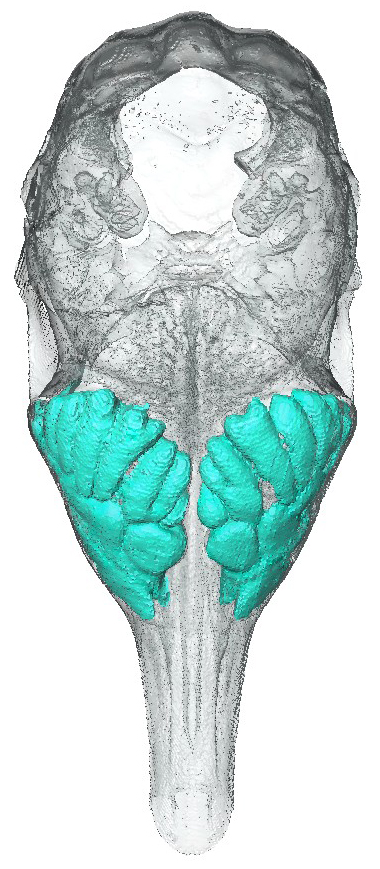

Supplement: Data S1 [file peerj-05-3593-s003.zip › all_sinuses_dorsal/LSU3244_juv_dors_transp.jpg]

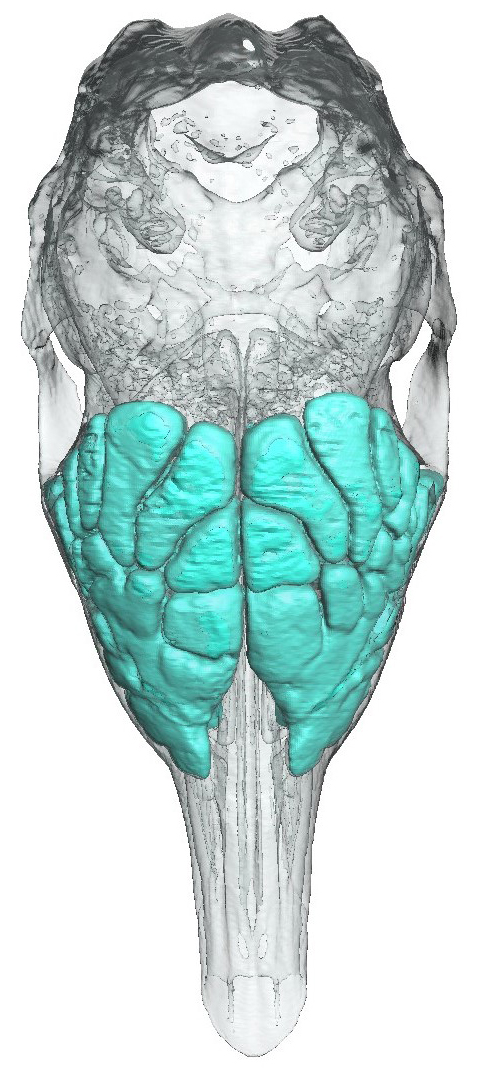

Supplement: Data S1 [file peerj-05-3593-s003.zip › all_sinuses_dorsal/179172_dorsal-trans.jpg]

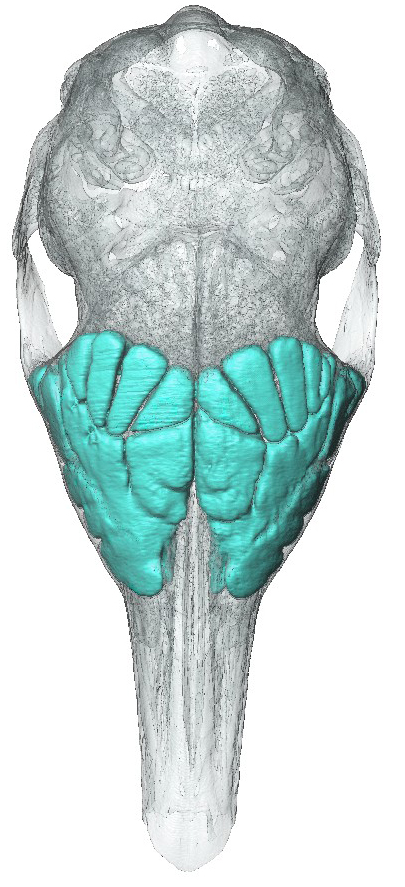

Supplement: Data S1 [file peerj-05-3593-s003.zip › all_sinuses_dorsal/Dasypus_usnm020920_dorsal_transp.jpg]

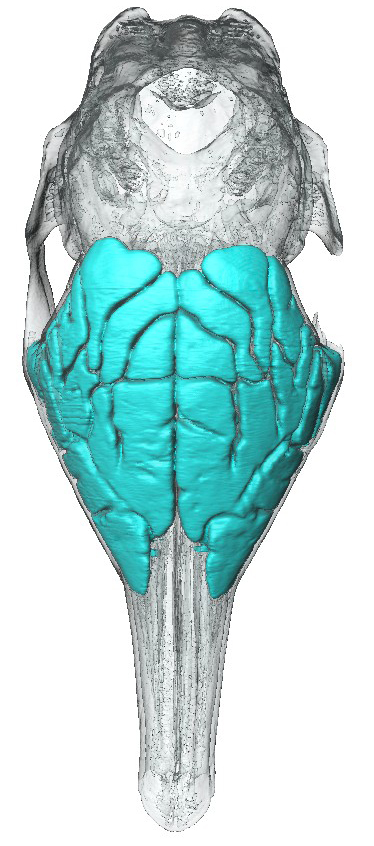

Supplement: Data S1 [file peerj-05-3593-s003.zip › all_sinuses_dorsal/LH_dasypus_nov_98-3-2-153_SnapshotB.jpg]

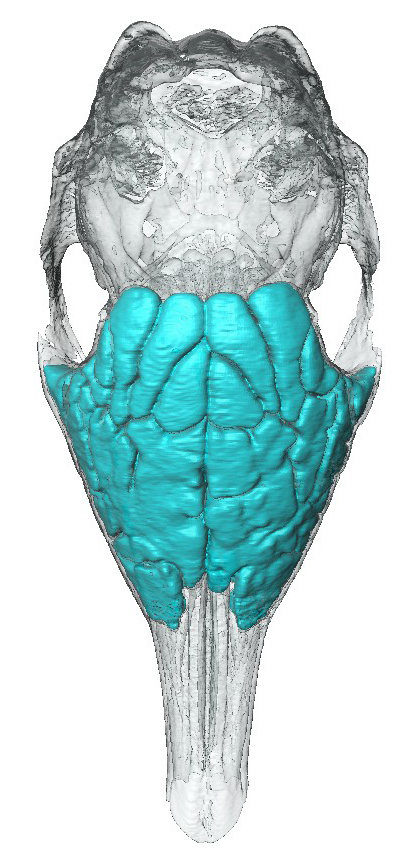

Supplement: Data S1 [file peerj-05-3593-s003.zip › all_sinuses_dorsal/dasypus-usnm337563_SnapshotB.jpg]

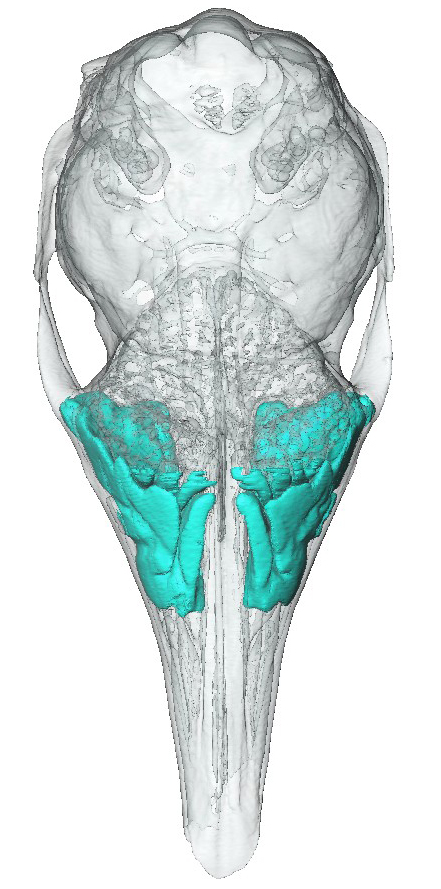

Supplement: Data S1 [file peerj-05-3593-s003.zip › all_sinuses_dorsal/Dasypus_sept_3-9-5-155_dors_transp_ORTHO.jpg]

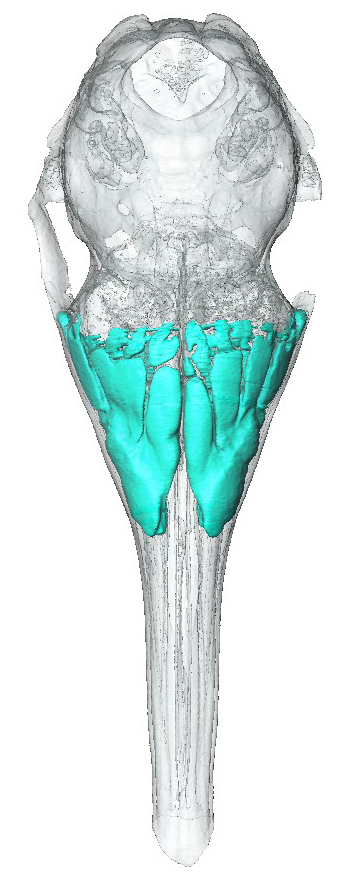

Supplement: Data S1 [file peerj-05-3593-s003.zip › all_sinuses_dorsal/das_pil_94-10-1-13_498-470-963_rec102um_dorsa_transp.jpg]

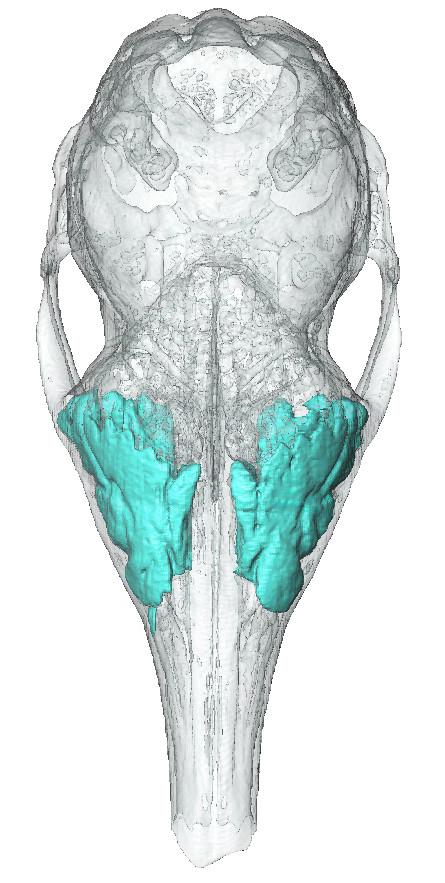

Supplement: Data S1 [file peerj-05-3593-s003.zip › all_sinuses_dorsal/Dasypus_hyb_amnh_205721_dorsal_transp2.jpg]

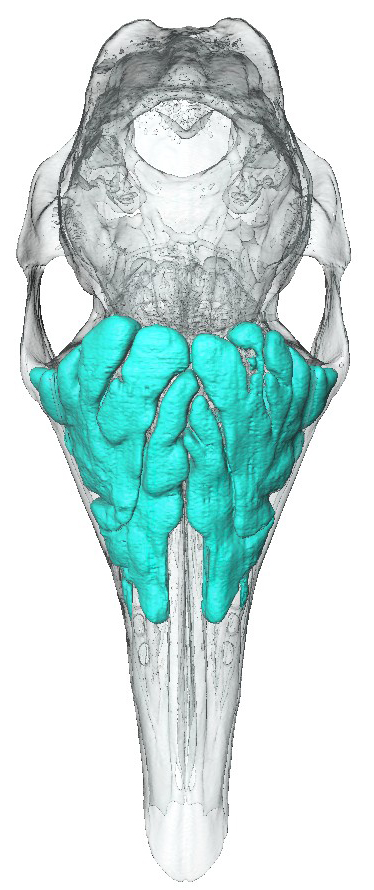

Supplement: Data S1 [file peerj-05-3593-s003.zip › all_sinuses_dorsal/Dasypus_kappleri_USNM388210_492_492_873_144μm_dorsal_transp.jpg]

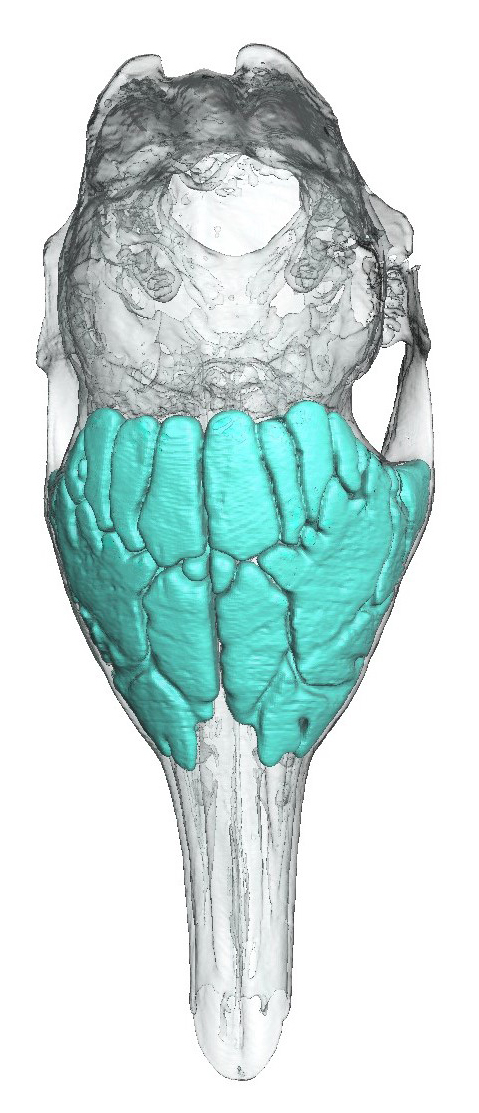

Supplement: Data S1 [file peerj-05-3593-s003.zip › all_sinuses_dorsal/171052_dorsal_trans.jpg]

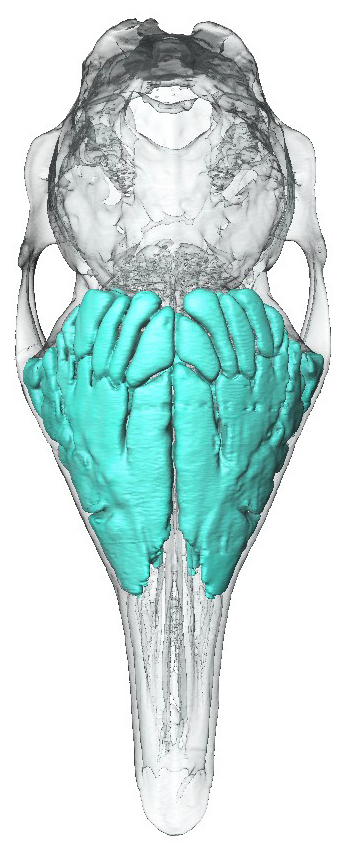

Supplement: Data S1 [file peerj-05-3593-s003.zip › all_sinuses_dorsal/LH_dasypus_nov_11-10-27-3-SnapshotB.jpg]

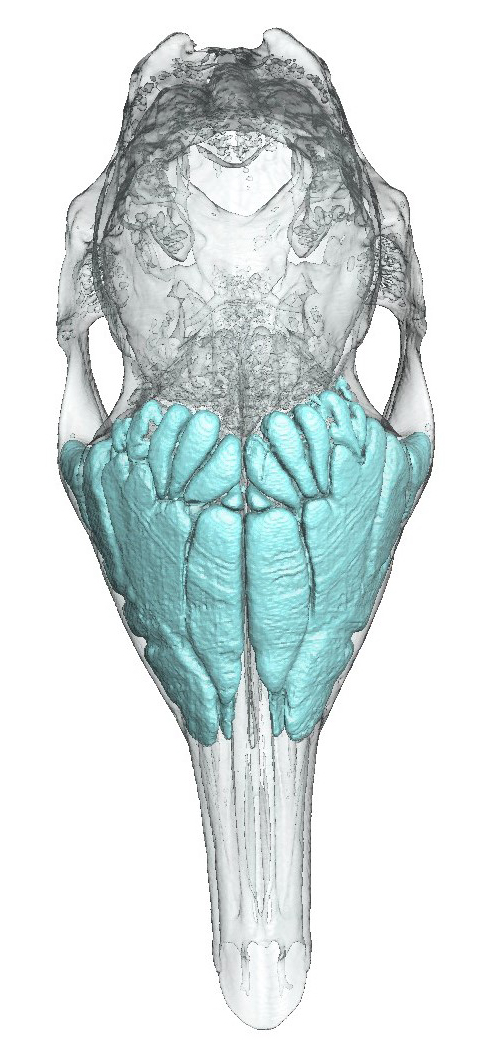

Supplement: Data S1 [file peerj-05-3593-s003.zip › all_sinuses_dorsal/LSU_12306_Peru_Loreto_dorsal_trans.jpg]

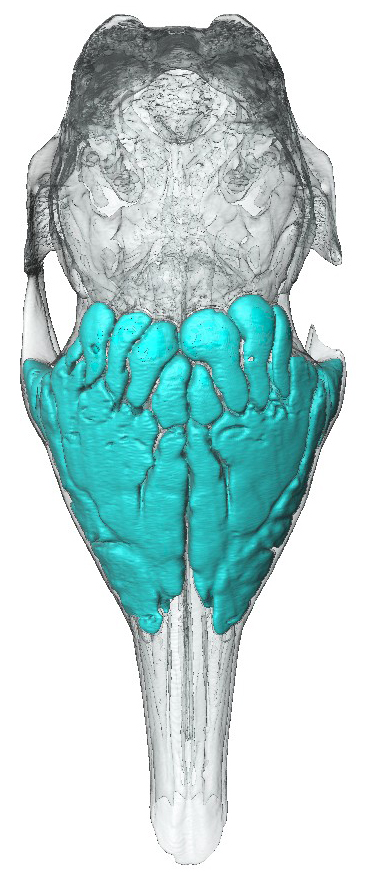

Supplement: Data S1 [file peerj-05-3593-s003.zip › all_sinuses_dorsal/LH_dasypus_nov_24-12-12-73_SnapshotB.jpg]

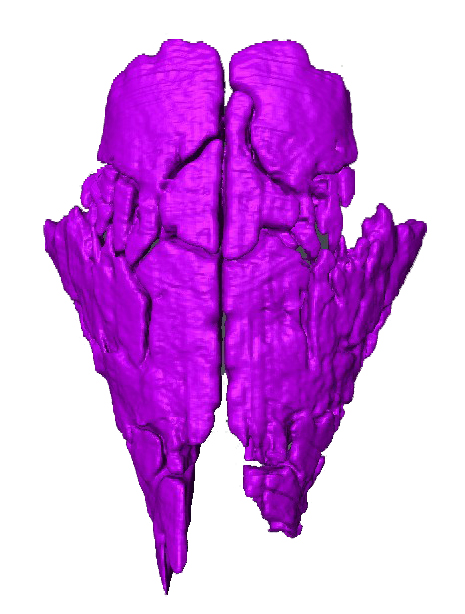

Supplement: Data S1 [file peerj-05-3593-s003.zip › all_sinuses_dorsal/das_nov_leiden_20958_suriname_femelle2.jpg]

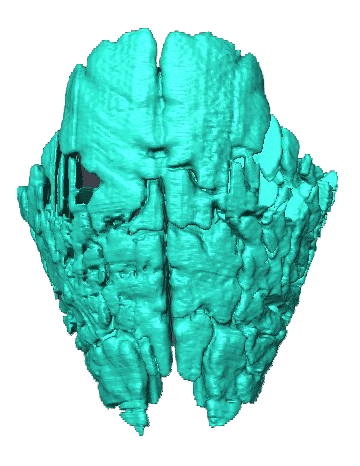

Supplement: Data S1 [file peerj-05-3593-s003.zip › all_sinuses_dorsal/das_nov_leiden_18024_suriname_male2.jpg]

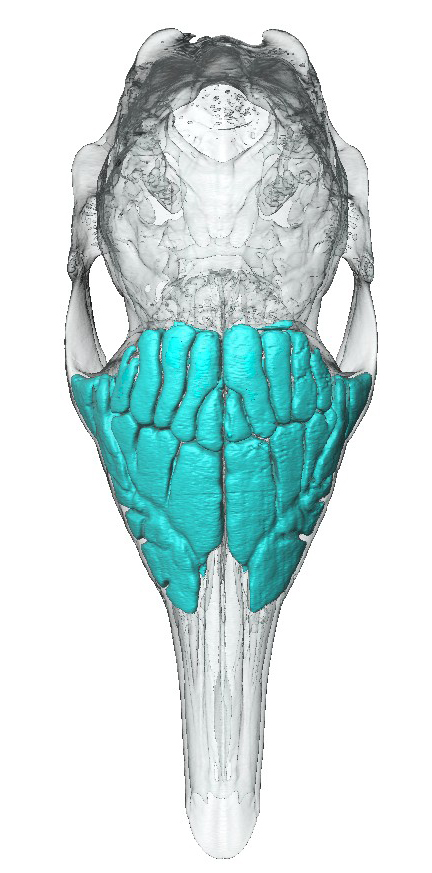

Supplement: Data S1 [file peerj-05-3593-s003.zip › all_sinuses_dorsal/AMNH205726_dorsal_transp.jpg]

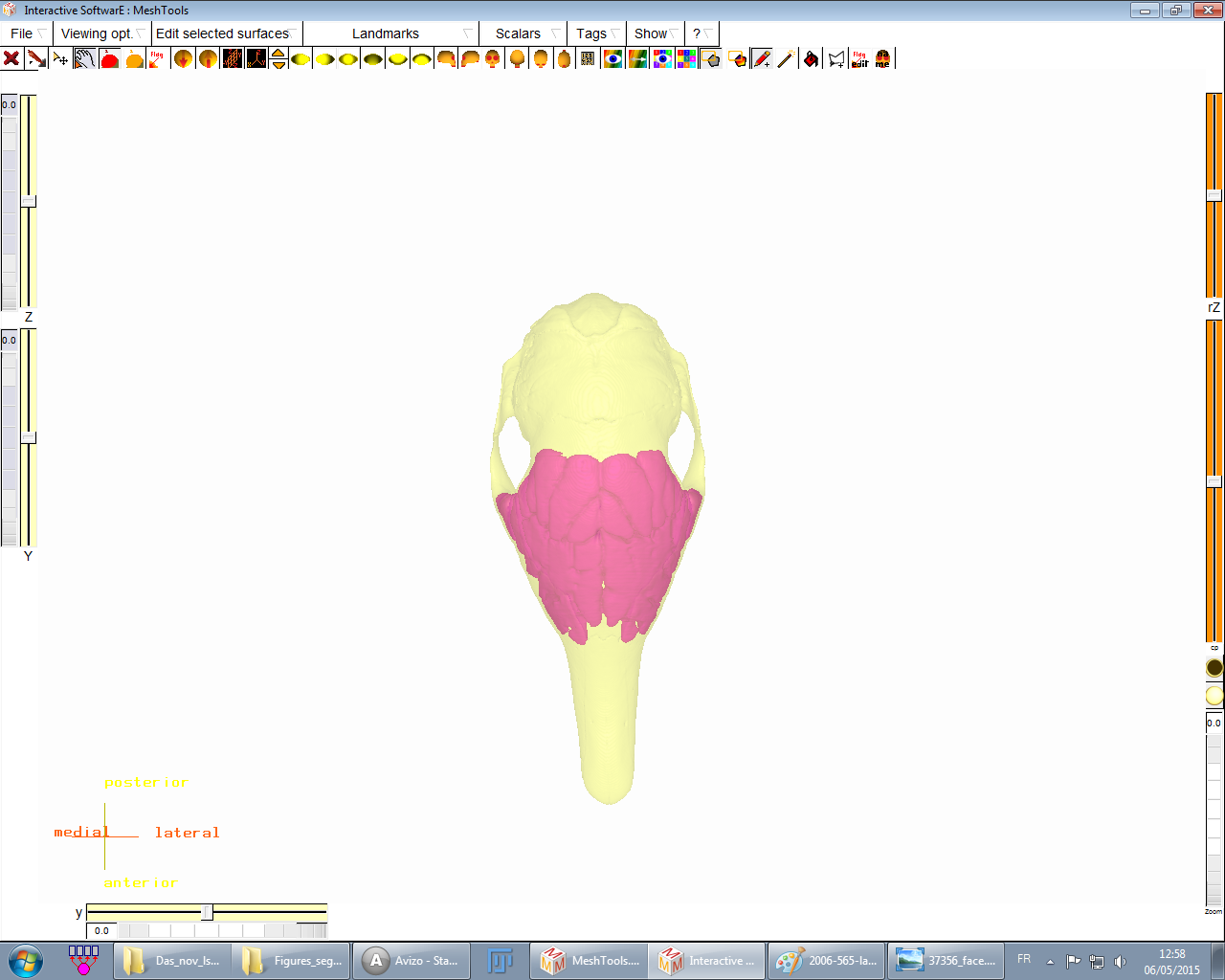

Supplement: Data S1 [file peerj-05-3593-s003.zip › all_sinuses_dorsal/29187-face.png]

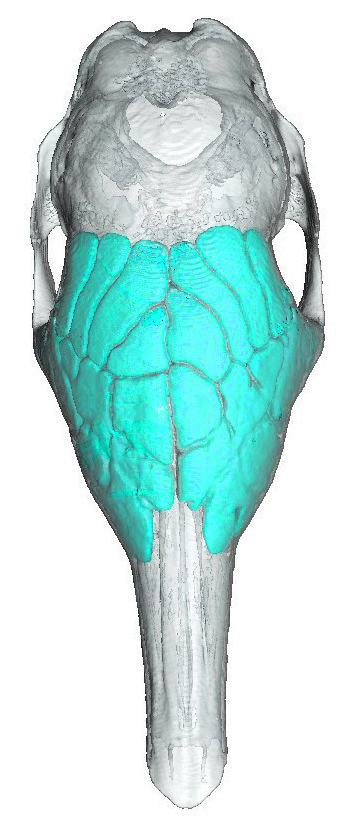

Supplement: Data S1 [file peerj-05-3593-s003.zip › all_sinuses_dorsal/31175_dorsal_trans4.jpg]

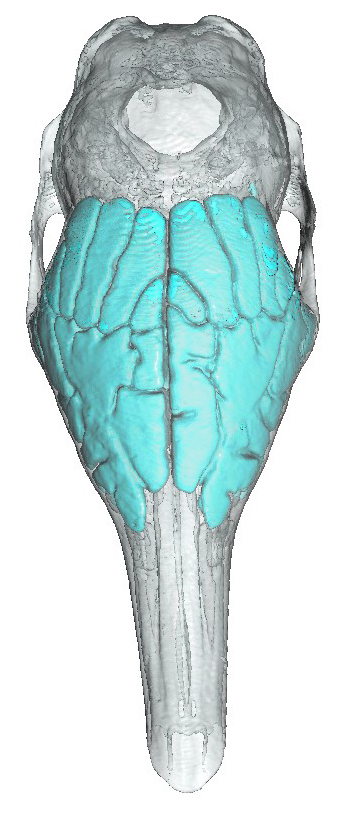

Supplement: Data S1 [file peerj-05-3593-s003.zip › all_sinuses_dorsal/29160_dorsal_trans4.jpg]

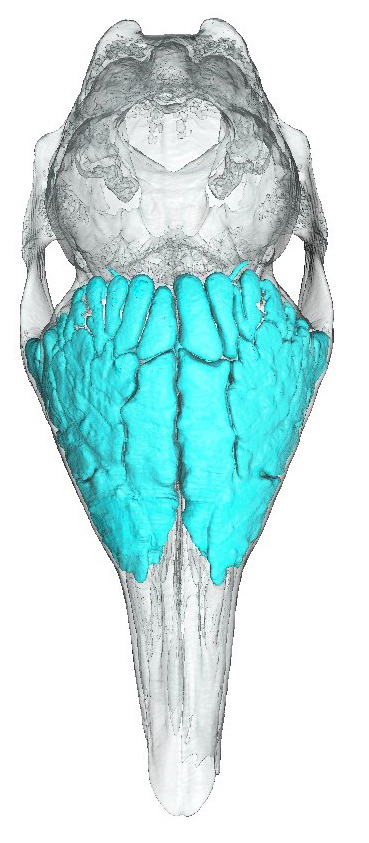

Supplement: Data S1 [file peerj-05-3593-s003.zip › all_sinuses_dorsal/usnm406700_dorsal_trans.jpg]

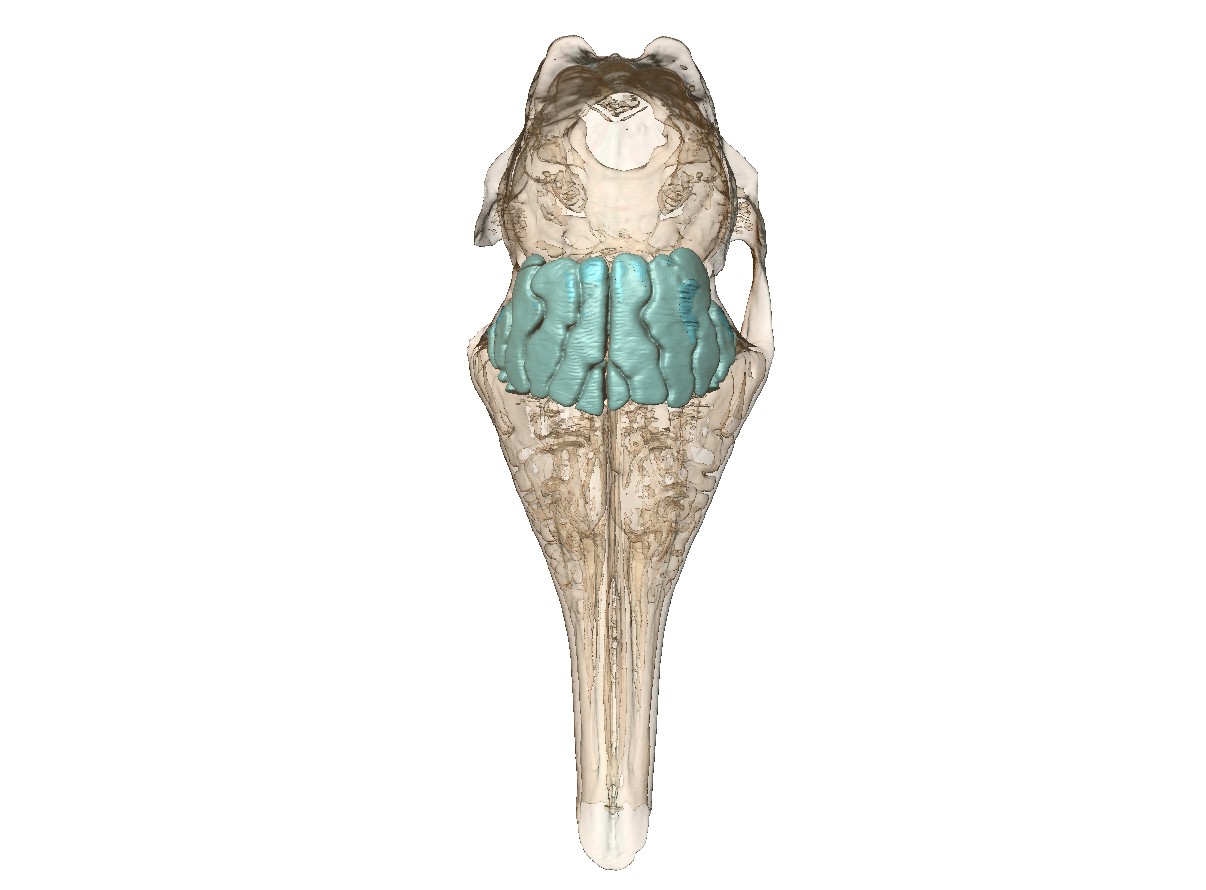

Supplement: Data S1 [file peerj-05-3593-s003.zip › all_sinuses_dorsal/LH_dasypus_nov5-7-5-21_Snapshot.jpg]
